# Disconnected Connections: How Insecure Attachment and Materialism Drive Phubbing Behaviors

**DOI:** 10.3390/bs16020216

**Published:** 2026-02-03

**Authors:** Phillip Ozimek, Esther Battenfeld, Elke Rohmann, Hans-Werner Bierhoff, Claire M. Hart, Rhia Perks, Carmen Surariu

**Affiliations:** 1 Department of Humanities, Vinzenz Pallotti University, 56179 Vallendar, Germany; 2 Department of Social Psychology, Faculty of Psychology, Ruhr University Bochum, 44801 Bochum, Germany; 3 Centre of Research on Self and Identity (CRSI), School of Psychology, University of Southampton, Southampton SO17 1BJ, UK; c.m.hart@soton.ac.uk (C.M.H.); c.surariu@soton.ac.uk (C.S.)

**Keywords:** attachment, attachment anxiety, attachment avoidance, phubbing, materialism

## Abstract

This study investigates the interplay between insecure attachment styles, materialism, and phubbing behaviors. Phubbing, the act of ignoring a partner in favor of smartphone use, is influenced by individual differences and societal norms. We hypothesized that attachment anxiety and avoidance would be positively associated with both enacted and perceived phubbing, and that materialism would mediate these relationships. Data were collected from 213 participants using validated scales for attachment, materialism, and phubbing. The results confirmed that attachment anxiety is positively associated with both enacted and perceived phubbing, while attachment avoidance is positively associated with perceived phubbing but not enacted phubbing. Materialism was found to mediate the relationship between attachment insecurity and phubbing behaviors. Specifically, materialism significantly mediated the positive relationships between attachment anxiety and both enacted and perceived phubbing, as well as between attachment avoidance and perceived phubbing. These findings suggest that materialistic values amplify the effects of insecure attachment on phubbing, highlighting the role of materialism as a compensatory mechanism for attachment-related insecurities. Future research should explore interventions targeting materialism and attachment anxiety to mitigate phubbing behaviors and improve relationship quality.

## 1. Introduction

Smartphones have become an indispensable part of daily life. They are constantly carried, serving a wide range of purposes, from navigating traffic, tracking health, staying connected through social media, messaging, video calls, etc. Smartphones are used in almost every situation, whether on the train, behind the wheel, at the dinner table, during social gatherings, at work, or while relaxing at home. In Germany, 85% of individuals aged 14 and older used mobile internet in 2023 (D21 Digital Index 2023/24: Annual Situation Report on the Digital Society, 2024), and 88.1% of private households owned at least one smartphone in 2022 (Destatis, 2022). Despite their ubiquitous presence, the appropriate use of smartphones remains ambiguously defined in society, leading to contrasting perceptions of when smartphone use is acceptable or inappropriate. This ambiguity has given rise to behaviors such as “phubbing”—the act of ignoring a partner in favor of smartphone use—posing new challenges in social relationships.

Phubbing is shaped by both personality traits and social norms. That is, individual differences and societal expectations influence how smartphone use is perceived—either as acceptable or inappropriate (Al-Saggaf & O’Donnell, 2019; Chotpitayasunondh & Douglas, 2016). Depending on the person and context, smartphone use may be seen as either socially acceptable or disrespectful. Phubbing is typically classified into two forms: enacted phubbing and perceived phubbing.

Enacted phubbing refers to the active behavior of using a smartphone during face-to-face interaction, where the individual is the subject performing the behavior. In contrast, perceived phubbing describes the experience of being phubbed—the individual is the object of the behavior. Importantly, these two perspectives do not always align: a person may engage in phubbing without intending to be rude, and the other may not perceive it negatively. Conversely, a person may not consider their own phone use inappropriate, yet the other party may still feel ignored or disrespected (Chotpitayasunondh & Douglas, 2016, 2018).

Since the term “phubbing” was first introduced in the Macquarie Dictionary in 2012, research in social psychology has explored its dynamics. Key drivers of enacted phubbing include reciprocity, where individuals phub in response to being phubbed (Al-Saggaf & O’Donnell, 2019; Thomas et al., 2022), and fear of missing out (FoMO), which compels individuals to check their phones frequently to stay connected to news and social updates (Al-Saggaf & O’Donnell, 2019; Schneider & Hitzfeld, 2021). FoMO, along with internet addiction and low self-control, has been shown to predict smartphone addiction, which in turn can lead to enacted phubbing (Chotpitayasunondh & Douglas, 2016; Karadag et al., 2015).

Enacted phubbing has been associated with several negative outcomes, including reduced conversational intimacy (Roberts & David, 2016; M. M. P. Vanden Abeele et al., 2016) and higher levels of depression (Al-Saggaf & O’Donnell, 2019; Ergün et al., 2020; Ivanova et al., 2020; X. Wang et al., 2017). Experimental studies suggest that phubbing disrupts the quality of interactions, mediated by unmet belonging needs (Chotpitayasunondh & Douglas, 2018). Additionally, enacted phubbing has been linked to increased anxiety, negative self-image, hostility, and loneliness (Ergün et al., 2020).

Beyond enacted phubbing, perceived phubbing also has significant psychological consequences. It often leads to feelings of social exclusion (Chotpitayasunondh & Douglas, 2018; David & Roberts, 2017), triggering a heightened need for attention and increasing social media use (David & Roberts, 2017). This cycle—feeling ignored, seeking attention, and intensifying social media engagement—can reduce overall well-being (David & Roberts, 2017).

Phubbing has been shown to be influenced by personality traits, particularly those tied to close relationship dynamics. For example, attachment theory, which describes individuals’ patterns of relating to others, offers a valuable lens for understanding phubbing. Individuals with insecure attachment styles are believed to be more prone to phubbing either as a form of emotional regulation or because they are more sensitive to perceived neglect (Bröning & Wartberg, 2022; Sun & Miller, 2023a). However, understanding the role of attachment anxiety and avoidance provides deeper insights into both enacted and perceived phubbing behaviors.

### 1.1. Attachment and Phubbing

Attachment theory offers a foundational framework for understanding individual differences in relationship behaviors and perceptions (Ainsworth et al., 1978; Bowlby, 1969, 1980; Mikulincer & Shaver, 2007). Two key dimensions of attachment—attachment anxiety and attachment avoidance—influence how individuals seek, interpret, and respond to interpersonal closeness and relational threats. These dimensions may help explain patterns in both enacted phubbing (one’s own behavior) and perceived phubbing (perception of others’ behavior).

Attachment anxiety is characterized by hyperactivation of the attachment system, driven by the fear of abandonment and the desire for closeness to a partner. Anxiously attached individuals frequently feel insufficiently loved and tend toward clingy behaviors (Neumann et al., 2007). Attachment avoidance, conversely, is marked by a deactivation of attachment behaviors to avoid emotional closeness and frustration. Avoidant individuals fear intimacy and trust, often prioritizing independence over emotional connection (Neumann et al., 2007).

While attachment styles are generally stable, changes can occur over time, influenced by shifts in relationship dynamics or the availability of attachment figures (Fraley & Dugan, 2021; Ross & Spinner, 2001). Attachment styles function as “working models,” adaptable to changes in relationship environments (Fraley & Dugan, 2021). Attachment security tends to improve in long-term relationships, and the attachment styles of partners interact, influencing each other in dyadic contexts (Bröning & Wartberg, 2022).

Meta-analytic findings reveal the negative effects of attachment anxiety and attachment avoidance on relationship quality. Attachment anxiety is linked to lower relationship satisfaction, greater emotional distress, and more destructive interactions (Li & Chan, 2012). In contrast, attachment avoidance is associated with dissatisfaction, emotional distance, and conflict avoidance (Li & Chan, 2012). These dimensions also interact with relationship quality, with attachment anxiety having a stronger impact on relationship conflict and attachment avoidance affecting relatedness and support (Li & Chan, 2012).

Attachment anxiety leads individuals to hypervigilantly monitor for signs of rejection or neglect (Bartholomew & Horowitz, 1991). Research supports that individuals with high attachment anxiety experience greater distress in response to partner unavailability and may ruminate about perceived neglect (Mikulincer et al., 2002). It is thus unsurprising that anxiously attached individuals are especially vulnerable to interpreting phubbing as rejection, given their heightened sensitivity to interpersonal threats (Gillath et al., 2005; Roberts & David, 2022). As such, research has shown that attachment anxiety is positively associated with perceived phubbing (Bröning & Wartberg, 2022; Carnelley et al., 2023). Again, mixed results have been found on the limited research examining attachment anxiety and enacted phubbing, with Carnelley et al. (2023) finding no association, and Sun and Miller (2023b) finding a positive association. These studies used different methods of data collection—a daily diary study versus a cross-sectional study which may account for these differences. The relationship with enacted phubbing may depend on situational factors. Anxious individuals may avoid phubbing to remain engaged with their partner, yet use their phone to seek validation when insecure, leading to complex, context-dependent relationships with enacted phubbing.

On the other hand, individuals with high attachment avoidance often value emotional distance and independence (Fraley & Shaver, 2000). For these individuals, enacted phubbing may serve as a disengagement strategy, using smartphones to avoid intimacy or emotional expression during interactions (Carnelley et al., 2023; Sun & Miller, 2023a). This behavior aligns with findings that avoidant individuals tend to prefer low self-disclosure and are less responsive to their partner’s bids for attention (Collins & Feeney, 2000; Wei et al., 2005). Findings on attachment avoidance and enacted phubbing however have been mixed, with one study showing no association (Carnelley et al., 2023) and another showing a positive association (Sun & Miller, 2023b). Perceived phubbing may not have the same negative impact on avoidant individuals, as their discomfort with closeness may reduce their sensitivity to feeling ignored or rejected (Mikulincer & Shaver, 2003). Research on avoidance and perceived partner phubbing has also yielded mixed results, with some studies reporting no significant association (Carnelley et al., 2023), while another found a gender-based difference—showing a positive link for men but a negative one for women (Bröning & Wartberg, 2022).

Understanding these dynamics contributes to the growing body of research on how digital communication technologies intersect with relational behaviors and attachment processes (Roberts & David, 2016; M. M. Vanden Abeele et al., 2019).

Based on the above, we tested the following hypotheses. Two layers of analysis were distinguished: (1) hypotheses on the dependencies between attachment variables and phubbing variables and (2) hypotheses on the mediation model which assumes that materialism mediates the influence of attachment variables on phubbing.

The first layer includes H1 to H4. The second layer includes H7 to H12. In addition, two hypotheses (H5 and H6) were derived on the connections between materialism and phubbing. The following hypotheses are located on the first layer:

**H1.**  *Attachment anxiety will be positively associated with perceived phubbing.*

Individuals high in attachment anxiety tend to be hypervigilant to signs of rejection or neglect in interpersonal interactions. As a result, they may be more likely to interpret others’ smartphone use during face-to-face encounters as exclusionary or dismissive, even when it is not intended that way.

**H2.**  *Attachment anxiety will be positively associated with enacted phubbing.*

Anxiously attached individuals may use smartphones during social interactions as a way to seek reassurance, validation, or connection through social media or messaging. This compensatory behavior may increase their likelihood of engaging in phubbing.

**H3.**  *Attachment avoidance will be positively associated with enacted phubbing.*

Avoidantly attached individuals tend to distance themselves emotionally from others and devalue interpersonal closeness. They may be more prone to disengaging from in-person interaction by turning to their smartphones, thus enacting phubbing.

**H4.**  *Attachment avoidance will be negatively or not significantly associated with perceived phubbing.*

The following hypotheses include materialism. Because in examining the links between attachment and phubbing, a relevant mediator that may underscore the relationship is materialism. Materialism, defined as the importance placed on possessions and their acquisition as a source of happiness or success (M. L. Richins & Dawson, 1992), is also linked to insecure attachment styles. This relationship may offer an explanatory mechanism for phubbing behaviors, which may serve as a compensatory strategy for individuals seeking external validation or security.

### 1.2. Materialism

Materialism is a concept with deep roots in psychological and sociological research. Broadly, materialism refers to the emphasis placed on possessions and the accumulation of material goods. It is often linked to the idea that material possessions fulfill deeper psychological needs. For instance, Maslow’s hierarchy of needs (Maslow, 1943) identifies basic needs such as water, food, and sleep, as well as security needs like physical safety and a stable home, as foundational for human well-being. Accumulating possessions is thought to provide greater security, ensuring survival and mitigating the risks of unforeseen events, such as job loss or natural disasters. A paid-off home, for example, may provide more security than renting in times of financial uncertainty.

Materialism is not just about satisfying physical needs, but about constructing and maintaining the self-image. According to Shrum et al. (2013), materialism involves the extent to which individuals seek to define themselves through the acquisition of possessions, experiences, and relationships that hold symbolic value. In this sense, “I am what I own,” or “I am what I experience” (Shrum et al., 2013), reflects the deeper connection between possessions and personal identity. This concept can extend to immaterial materialism, where intangible assets—such as social media presence, experiences, or relationships—serve as symbols of self-worth, much like physical possessions.

In today’s digital world, where individuals often present curated versions of themselves, immaterial items—such as the number of followers, edited photos, or status updates—serve as important symbols of status. These digital representations are akin to traditional material possessions, signaling social position and self-worth. Social comparisons are intrinsic to materialism, as individuals often measure their success against their peers, not just in terms of what they have, but how they compare to others in their social circle (Chan & Prendergast, 2007).

The pursuit of more possessions, experiences, or relationships is often insatiable. This endless pursuit of status, success, and material wealth can appear superficial and unsustainable, especially when it detracts from personal fulfillment (M. Richins & Fournier, 1991). Materialism has been linked to negative outcomes, such as reduced environmental concern (Segev et al., 2015), diminished prosocial behavior, and lower social well-being (Moldes & Ku, 2020).

Empirical evidence highlights the negative consequences of materialism: it is associated with lower well-being (Dittmar et al., 2014; Dittmar & Isham, 2022; Moldes & Ku, 2020) and life satisfaction (Ozimek et al., 2024). While materialism directly decreases consumer satisfaction, its indirect effect—mediated by self-improvement and competitiveness—can be positive (Thyroff & Kilbourne, 2018). Longmire et al. (2021) found that materialism can offer symbolic security during periods of anxiety, though this security is fleeting and does not alleviate objective fear. In uncertain times, materialism may be a coping mechanism to enhance self-esteem (Belk, 2015), particularly when opportunities for social advancement exist (Cho et al., 2016; Ger & Belk, 1996; X. Wang et al., 2022).

The relationship between materialism and self-image insecurity has also been well-documented. At a young age, individuals may develop materialistic tendencies as a way to cope with self-uncertainty. As individuals age and their self-image solidifies, materialism tends to diminish (Martin et al., 2019). Self-uncertainty, mediated by materialism, is associated with lower subjective well-being (B. Chang et al., 2023). This underscores the role of materialism as a response to both social insecurity and internal uncertainty (L. Chang & Arkin, 2002).

P. Wang et al. (2020) found that smartphone addiction is positively correlated with materialism in adolescents, with materialists using social media for social comparison and objectifying their friends as digital possessions (Ozimek et al., 2017). This tendency to objectify others could contribute to phubbing behavior, as individuals with high materialistic values may focus more on the validation they get from their devices than the emotional connection with those present in the room.

Despite the wealth of research on materialism, there is limited exploration of its relationship with enacted phubbing. However, constructs related to phubbing—such as social media use—have been explored. Ozimek et al. (2024) found that materialism is positively associated with social comparison orientation, leading to passive social media use and even social media addiction. Social media use encourages upward social comparisons (Schmuck et al., 2019), where individuals measure their status against others, often by showcasing possessions or experiences.

Despite the limited research in this area, we hypothesize the following:

**H5.**  *Materialistic values will be positively associated with enacted phubbing.*

Individuals with high levels of materialism tend to be more phone-dependent, using their devices for various activities like online shopping or status signaling (e.g., showcasing luxury brand technology). This heavy reliance on smartphones for materialistic goals can lead to increased enacted phubbing, as digital engagement often takes precedence over face-to-face interactions. Smartphones, in this sense, become tools for maintaining materialistic goals, such as tracking purchases or projecting curated online identities, further diminishing in-person connection.

**H6.**  *Materialistic values will be positively associated with perceived phubbing.*

For materialistic individuals, perceived phubbing may become more pronounced. They may interpret others’ phone use during social interactions as a sign of disinterest or as a reflection of a shifting value system—in so far that the partner is more interested in social media and consumption than in the romantic relationship. This interpretation of smartphone use as a threat to social connection can amplify the perception of being phubbed.

Given the links between materialism and social comparison, it is plausible that individuals high in attachment anxiety may engage in materialistic behaviors to compensate for feelings of insecurity. Such individuals may define their self-worth through status and social comparison, making materialism a key strategy to affirm their value. Consequently, enacted phubbing could be an expression of their need to seek status, as they turn to their smartphones during in-person interactions to validate themselves through digital engagement. We examine the novel relationships between attachment, materialism and phubbing below.

### 1.3. Attachment, Materialism, and Phubbing

Anxiously attached individuals often experience chronic insecurity and a low sense of self-worth, which can lead them to seek external validation. In this context, material possessions can serve as compensatory mechanisms to counteract feelings of rejection and insecurity (Mikulincer & Shaver, 2007). These individuals may view possessions to boost self-esteem or secure social approval. As a result, materialistic values may be adopted as a strategy for identity formation and self-worth enhancement. In contrast, avoidantly attached individuals tend to devalue emotional closeness and often prioritize autonomy and control. This can lead them to seek comfort in non-relational sources, such as material possessions or status symbols (Kasser et al., 2004).

Materialism in this case functions to fulfill emotional needs and creates a sense of self-worth without relying on others. Possessions provide a tangible source of validation, allowing these individuals to maintain their sense of independence while also enhancing their social status.

Based on materialism as a mediator we hypothesized on the second layer of dependencies the following propositions:

**H7.**  *Attachment anxiety will be positively associated with materialistic values.*

**H8.**  *Attachment avoidance will be positively associated with materialistic values.*

As far as we know, we are the first to explore the role of materialism as a mediator in explaining how attachment influences phubbing. We hypothesized the following:

**H9.**  *The relationship between attachment avoidance and enacted phubbing will be mediated by materialism.*

Individuals high in attachment avoidance tend to prioritize independence and self-sufficiency. However, a materialistic orientation may push them to rely more heavily on digital engagement, as material possessions and status are often showcased through smartphones. This increased reliance on devices for status signaling may lead to greater enacted phubbing in social settings, as materialistic goals take precedence over interpersonal goals.

**H10.**  *The relationship between attachment anxiety and enacted phubbing will be mediated by materialism.*

For anxiously attached individuals, material possessions and digital technology (especially smartphones) often become tools for self-validation and social affirmation. This materialistic orientation can lead to increased phone use during social interactions, as individuals attempt to boost their self-worth through external validation. Consequently, this behavioral tendency is likely to result in higher levels of enacted phubbing, where the digital world eclipses the present moment with others.

**H11.**  *The relationship between attachment anxiety and perceived phubbing will be mediated by materialism.*

Anxiously attached individuals may place heightened importance on appearance, which is related to cues and material possessions, to feel secure and accepted. As a result, they are more likely to interpret others’ smartphone use as a threat to their social value. This heightened sensitivity can lead to increased perceptions of being phubbed, particularly in situations where individuals feel their self-worth is being undermined by others’ digital preoccupation.

**H12.**  *The relationship between attachment avoidance and perceived phubbing will be mediated by materialism.*

Although avoidantly attached individuals often suppress their interpersonal needs, they may still be attuned to status-related cues, including the subtle messages conveyed through smartphones. A materialistic orientation may heighten their awareness of these cues, increasing their likelihood of perceiving phubbing (especially in contexts where social comparison is salient, such as during interactions that involve visible displays of status or possessions).

## 2. Materials and Methods

### 2.1. Participants

We recruited 213 participants using a snowball sampling technique. Of these, 72.3% were female, 27.2% were male, and 0.5% preferred not to disclose their gender. The participants had an average age of 34.53 years (median = 27), with ages ranging from 18 to 71 years. The highest level of education stated was secondary school certificate 6.1%, vocational baccalaureate 10.3%, high school diploma 54.5%, and academic degree 28.6%. A majority (73.2%) of participants were students, and 47.9% were studying psychology. Additionally, 75.1% of participants were employed. The average duration of participants’ romantic relationship was 11.5 years (median = 4.75 years), with a minimum relationship duration of 3 months and a maximum of 55.75 years. A total of 67.4% of participants lived with their partner, and. 39.1% were married. Furthermore, 60.1% of participants reported seeing their partner very often (several times a day). On average, participants spent 2.36 h per day on social media (*SD* = 1.59).

### 2.2. Design

The data were collected online via a questionnaire. The average completion time was approximately one hour. The prerequisites for participation were being 18 years of age or older, having been in a romantic relationship for at least three months, and regularly using at least one social media platform (at least once a month). At the beginning of the survey, participants were informed about the purpose of the study and provided their consent to take part in the study. There was no deception, nor were any variables manipulated. The study was approved by the local ethics committee at the Ruhr University Bochum (protocol code 595). The survey took part in a larger study with respect to phubbing behavior in collaboration between the Ruhr University Bochum, the Vinzenz Pallotti University, and the University of Southhampton. There were no incentives. We described the measured used in the following sections. They are presented in a randomized order.

### 2.3. Measures

#### 2.3.1. Attachment

Attachment was assessed using the Experience in Close Relationships Scale, German 10-item version (Neumann et al., 2023). This scale, validated by Neumann et al. (2023), is a short-form adaption (10-items) of the original 36-item scale (Brennan et al., 1998; Neumann et al., 2007). The questionnaire measures attachment anxiety and attachment avoidance, each with five items, using a 7-point rating scale from 1 (strongly disagree) to 7 (strongly agree). Sample items include “I talk to my partner about almost everything” (indicating low avoidance) and “I need confirmation that my partner loves me” (indicating high anxiety). In the current study, the internal consistency was adequate, with attachment avoidance α = 0.82 and attachment anxiety α = 0.75.

#### 2.3.2. Materialism

Materialism was assessed using the German version of the Material Values Scale (G-MVS; Müller et al., 2013), developed by M. L. Richins (2004). The original scale consists of 15 items that load on three factors: Centrality, Success, and Happiness. The German version (Müller et al., 2013) was validated using a representative sample (*N* = 2295) and showed high internal reliability; α = 0.89 for the overall 15-item scale. Regarding construct validity, 52 patients with shopping addiction and 347 students completed the G-MVS, the Compulsive Buying Scale (Faber & O’Guinn, 1992), and the depression scale of the Patient Health Questionnaire (Kroenke et al., 2009). Supporting the construct validity of the scale, the findings confirmed that the G-MVS correlated positively with compulsive buying, but not with depression. This pattern of results was also observed in the original English version of the scale (M. L. Richins, 2004).

Participants indicated their level of agreement with the scale items using a 5-point rating scale, from 1 (strongly disagree) to 5 (strongly agree). Example items include “My life would be better if I owned certain things that I do not yet have” and ‘I admire people who have expensive houses, cars and clothes’. In the current study, the scale demonstrated excellent reliability (α = 0.90).

#### 2.3.3. Enacted Phubbing

Enacted phubbing was assessed using a German translation of the Phubbing Scale by Karadag et al. (2015). The scale was translated into German by our team and independently back-translated. Differences between the original and translated versions were reviewed, and the team reached a consensus on the best German phrasing.

The original scale, developed using data from focus groups, consists of ten items and has a reported reliability between α = 0.87. The German translation consists of ten items, measured on a 5-point rating scale, from 1 (never) to 5 (always). Participants were instructed to indicate how often certain behaviors applied to them. Example items are “My eyes start wandering on my phone when I’m together with others” and” I feel incomplete without my mobile phone.” For an overview, see Table A1 in the Appendix A. In the German version, the scale demonstrated adequate reliability (α = 0.75).

#### 2.3.4. Perceived Phubbing

To measure phubbing perceptions in partnerships, we used the Partner Phubbing Scale by David and Roberts (2020). This scale, originally designed to assess general social interactions, was translated into German by our team and then independently back-translated. The questionnaire consists of nine items measuring phubbing perception (e.g., “My partner places his or her cell phone where they can see it when we are together.”). Items were rated on a 5-point rating scale from 1 (never) to 5 (always). For this study, the wording of the items was adapted from general social interactions (as in the original version) to specifically refer to romantic partnerships. For an overview, see Table A2 in Appendix A. The Partner Phubbing Scale demonstrated good reliability in the present study (α = 0.85).

### 2.4. Statistical Analyses

Statistical analysis was conducted using SPSS 29. To ensure comparability of effects, mean values of the scales and subscales were calculated. PROCESS 4 Beta (Hayes, 2022) was used for the mediation analyses. The prerequisites for mediation analysis include linearity, normal distribution of residuals, homoscedasticity, independence, and temporal precedence (Hayes, 2022). Since PROCESS 4 beta employs bootstrapping, it is robust regarding the distribution of the residuals and homoscedasticity. Independence of residuals was ensured by the research design. However, temporal precedence was not established by the research design, as cross-sectional data were used. A summary of all model assumption checks can be found at: https://osf.io/t46x7/overview?view_only=c232217761194b91bcd9593b30f996c9, accessed on 30 November 2025. Although multiple correlations were computed for hypothesis testing (H1–H8), the primary conclusions of this study are based on mediation analyses (H9–H12) estimated with bootstrapped confidence intervals, which provide robust inference for indirect effects; moreover, post hoc robustness checks did not indicate systematic bias or patterns consistent with inflated significance.

## 3. Results

### 3.1. Descriptive Results

Participants exhibited moderate levels of attachment anxiety (*M* = 3.62, *SD* = 1.69) and relatively low levels of attachment avoidance (*M* = 2.28, *SD* = 1.17, range = 1–7). Regarding materialistic values, participants scored moderately on average (*M* = 2.36, *SD* = 0.79; range = 1–5). For both enacted phubbing and perceived phubbing, participants exhibited moderate scores (enacted phubbing: *M* = 2.46, *SD* = 0.59; perceived phubbing: *M* = 2.60, *SD* = 0.73; range = 1–5).

### 3.2. Correlations

Hypotheses 1–8 were tested using product–moment correlations (see Table 1). Apart from H3, which was refuted due to a nonsignificant correlation (attachment avoidance was not positively correlated with enacted phubbing), all hypotheses were supported. Specifically, attachment anxiety was positively associated with perceived phubbing (H1), attachment anxiety was positively associated with enacted phubbing (H2), attachment avoidance was positively associated with perceived phubbing (H4), materialistic values and enacted phubbing were positively correlated (H5), and materialistic values and perceived phubbing were positively correlated (H6). In addition, attachment anxiety was positively associated with materialistic values (H7) and attachment avoidance was positively associated with materialistic values (H8).

### 3.3. Mediation Analyses

**Figure 1:**
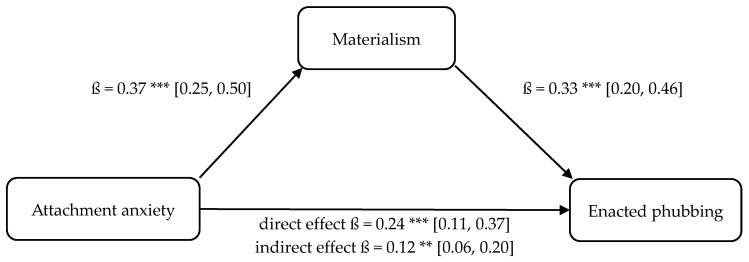
Mediation model 1: attachment anxiety (UV), materialism (M), phubbing behavior (AV). Note. Bootstrapped confidence intervals in brackets. ** *p* < 0.01, *** *p* < 0.001.

**Figure 2:**
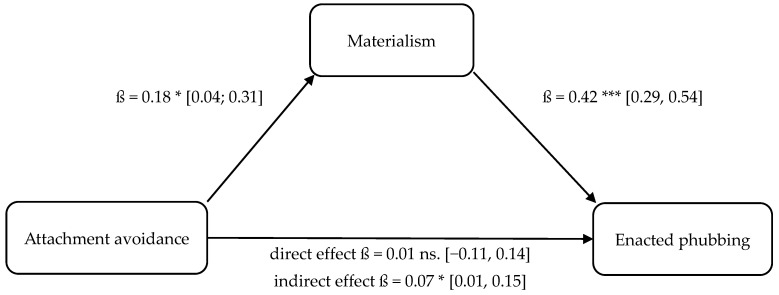
Mediation model 2: attachment avoidance (UV), materialism (M), enacted phubbing (AV). Note. Bootstrapped confidence intervals in brackets. * *p* < 0.05, *** *p* < 0.001; ns = not significant.

**Figure 3:**
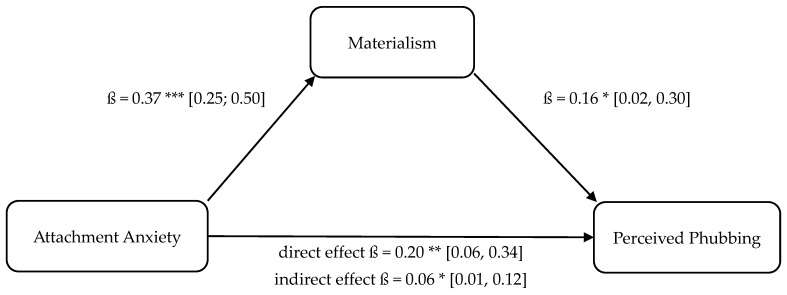
Mediation model 3: attachment anxiety (UV), materialism (M), perceived phubbing (AV). Note. Bootstrapped confidence intervals in brackets. * *p* < 0.05, ** *p* < 0.01, *** *p* < 0.001.

**Figure 4:**
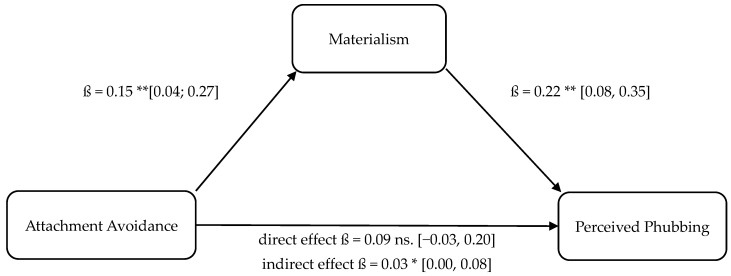
Mediation model 4: attachment avoidance (UV), materialism (M), perceived phubbing (AV). Note. Bootstrapped confidence intervals in brackets. * *p* < 0.05, ** *p* < 0.01; ns = not significant.

Four separate mediation analyses were conducted to test Hypotheses 9–12. These models tested whether the relationship between attachment insecurity (anxiety, avoidance) and phubbing (enacted, perceived) is mediated by materialism. These results are illustrated in Figure 1, Figure 2, Figure 3 and Figure 4.

The statistical analyses using both OLS regression and the determination of bootstrapping confidence intervals confirmed the assumed indirect mediation paths. The positive relationship between attachment anxiety and enacted phubbing (cf., H10 and mediation 1, respectively; partial mediation) as well as the positive relationship between attachment avoidance and enacted phubbing (cf., H9 and mediation 2, respectively; full mediation) were significantly mediated indirectly via materialism. Furthermore, the positive relationship between attachment anxiety and perceived phubbing (cf., H11 and mediation 3, respectively; partial mediation) as well as the positive relationship between attachment avoidance and perceived phubbing (cf., H12 and mediation 4, respectively; full mediation) were significantly indirectly mediated by materialism, confirming hypotheses H11 and H12.

### 3.4. Replicability and Post Hoc Power

We checked our results using the p-checker app (https://shinyapps.org/apps/p-checker/ (accessed on 30 November 2025)) and calculated a median observed post hoc power of 0.9911. Above, we found a replicability index of R = 0.9821, showing that our results are around 98% replicable. A test of insufficient variance (TIVA) shows that no biases occurred (Chi square = 33.283, *p* > 0.05).

## 4. Discussion

This research breaks new ground by relating phubbing, a recently emerging topic in social media research, to attachment insecurity (i.e., attachment anxiety and attachment avoidance) and materialism as a personality-based value orientation. In addition, the present findings demonstrate that materialism mediates key associations between attachment insecurity and both enacted and perceived phubbing. Thus, the guiding research idea was not only that materialism follows from attachment insecurity, but that materialism passes along the effects of attachment insecurity on phubbing.

### 4.1. Direct Associations Between Attachment and Phubbing (H1–H4)

From the perspective of attachment theory (Bowlby, 1969, 1980; Mikulincer & Shaver, 2007), attachment anxiety and attachment avoidance represent basic dimensions of social bonding that shape how individuals regulate closeness, respond to relational threat, and interpret partner behavior. Accordingly, the direct associations between attachment variables and phubbing (H1–H4) provide a foundational framework for understanding phubbing as an interpersonal phenomenon rather than merely a technology use habit.

The confirmation of H1 (attachment anxiety is positively associated with perceived phubbing) corresponds with the proposition that anxiously attached individuals are hypervigilant to signs of rejection or unavailability. Given that smartphone engagement in face-to-face interaction can signal reduced responsiveness, anxiously attached individuals may be particularly likely to experience partner phone use as exclusionary or dismissive. This interpretation aligns with the hyperactivation of the attachment system, where ambiguous cues may be perceived as threatening to relationship security.

In addition, the confirmation of H2 (attachment anxiety is positively associated with enacted phubbing) suggests that anxious attachment not only shapes perception but also relates to actual smartphone-related interaction behavior. A conceptual implication is that enacted phubbing may partly reflect affect regulation or reassurance seeking. In other words, anxiously attached individuals may use their smartphone to reduce insecurity (e.g., by seeking validation, maintaining online contact, or monitoring social information), thereby increasing the likelihood of phubbing behaviors during social interaction.

In contrast, H3 (attachment avoidance is positively associated with enacted phubbing) was refuted by the nonsignificant correlation between attachment avoidance and enacted phubbing. Although the association was in the expected direction, this refutation may have several explanations. First, it may reflect measurement-related issues or sample-specific characteristics that reduce the observable link between avoidance and enacted phubbing. Second, it is also conceivable that attachment avoidance does not directly elicit enacted phubbing as a stable behavioral tendency. Avoidantly attached individuals may disengage from interpersonal interaction via other strategies (e.g., reduced emotional disclosure, withdrawal, or de-emphasizing closeness) rather than smartphone-related disengagement. Thus, the absence of a direct association in the present data does not necessarily imply that avoidance is unrelated to disengagement, but that enacted phubbing may not be the primary behavioral expression of avoidant attachment in this context. Given the mixed evidence in prior work, additional data collection seems desirable to clarify whether a robust association between avoidance and enacted phubbing should be expected.

Finally, the confirmation of H4 (attachment avoidance is positively associated with perceived phubbing) indicates that avoidance may still be relevant for phubbing experiences, particularly on the perceptual level. This finding is noteworthy because avoidant attachment is sometimes assumed to reduce sensitivity to interpersonal threats. However, perceived phubbing may not only signal rejection, but also disrupt expected interaction scripts and communicative flow. Thus, even avoidantly attached individuals may register and report phubbing perceptions, potentially because partner phone use interferes with autonomy, predictability, or interaction quality. In sum, the results of the direct effects (H1, H2, and H4) reveal a basic network of relationships between social bonding and phubbing.

A central theoretical contribution of the present study lies in identifying materialism as a psychologically meaningful mediator between attachment insecurity and phubbing. Although prior research has linked phubbing to general patterns of technology use and social media engagement, the current findings go beyond this descriptive level by demonstrating that phubbing can be understood as a value-driven and relationally embedded behavior. In line with attachment theory (Bowlby, 1969, 1980; Mikulincer & Shaver, 2007), insecure attachment is associated with chronic concerns about relational security and availability, which motivate compensatory regulatory strategies. Our results suggest that materialistic value orientations provide one such compensatory pathway, translating attachment-related insecurity into attentional and behavioral shifts toward smartphones during interpersonal interactions.

Materialism is particularly well suited as an explanatory construct because it does not merely reflect an interest in technology or media use per se, but rather a broader orientation toward external objects and symbols as sources of security, control, and self-worth. From this perspective, smartphones and digitally mediated information can acquire symbolic value, functioning as readily available resources that promise immediacy, reassurance, and perceived relevance. Phubbing thus emerges as a behavioral manifestation of materialistic meaning-making in social contexts, where attention is directed toward potentially “valuable” digital content at the expense of face-to-face interaction. The substantial associations between materialism and enacted phubbing (cf., H7), as well as the mediation effects observed in H9–H12, underscore the relevance of this interpretation.

Importantly, positioning materialism as a mediator also clarifies why attachment insecurity relates to phubbing beyond general technology use. The mediation analyses demonstrate that attachment anxiety and attachment avoidance influence phubbing not only directly (H1, H2, H4), but also indirectly through materialistic value orientations. This pattern highlights materialism as a central explanatory mechanism that connects relational insecurity with everyday digital behavior and extends existing theoretical models of phubbing by embedding them in broader motivational and value-based frameworks.

### 4.2. Materialism and Phubbing (H5–H6)

The next set of hypotheses focused on materialism as a value orientation and its associations with enacted and perceived phubbing. The confirmation of H5 (materialism is positively associated with enacted phubbing) and H6 (materialism is positively associated with perceived phubbing) supports the proposition that materialistic values overlap with phubbing in meaningful ways.

Materialism reflects the importance placed on possessions and their acquisition as sources of success and happiness (M. L. Richins & Dawson, 1992). In addition, more recent conceptualizations highlight materialism as a form of identity goal pursuit and symbolic self-definition (“I am what I own” or “I am what I experience”; Shrum et al., 2013). In the digital age, smartphones provide constant access to social comparison, consumption-related activities, curated self-presentation, and social reinforcement. Thus, smartphones may function as tools through which materialistic goals and status-relevant information can be monitored and pursued. Conceptually, this implies that enacted phubbing may be more likely when the smartphone is experienced as a psychologically central object for reinforcement and identity maintenance. Moreover, materialistic individuals may interpret partner phone use as a sign of shifting value priorities (e.g., attention invested in digital status and consumption rather than interpersonal connection), which may foster perceived phubbing.

### 4.3. Attachment and Materialism (H7–H8)

The present study also assumed that attachment insecurity is positively associated with materialism. Both H7 (attachment anxiety is positively associated with materialism) and H8 (attachment avoidance is positively associated with materialism) were confirmed. These findings are consistent with theoretical views that materialism can serve compensatory functions under conditions of insecurity. Anxiously attached individuals often experience chronic doubts about relational worth and acceptance, which can motivate external validation seeking. Material possessions or status-related symbols may provide a temporary sense of self-worth and social approval. Avoidantly attached individuals, in turn, tend to rely less on relational closeness and may prioritize autonomy and self-sufficiency. In this case, materialism can offer a non-relational pathway to maintain control, status, and self-definition without reliance on interpersonal dependency.

Thus, from a conceptual viewpoint, insecure attachment may increase the attractiveness of materialistic goal pursuit because it offers a symbolic route to security, self-enhancement, and stability.

### 4.4. Materialism as Mediator Between Attachment Insecurity and Phubbing (H9–H12)

The central theoretical contribution of the present study concerns the proposition that materialism mediates the relationship between attachment insecurity and phubbing. As far as we know, this is among the first studies to examine materialism as a mediator in explaining how attachment influences enacted and perceived phubbing. The mediation results were consistent and support the research idea that materialism functions as a key “turntable” within the network of attachment and phubbing variables.

Regarding enacted phubbing, the relationship between attachment anxiety and enacted phubbing was significantly mediated by materialism, confirming H10 (partial mediation). This pattern suggests that anxious attachment contributes to enacted phubbing not only directly (as in H2), but also indirectly through increased materialistic values. In other words, attachment anxiety may foster a value orientation toward external validation and status-related goals, which may increase smartphone engagement and ultimately enacted phubbing.

Furthermore, the relationship between attachment avoidance and enacted phubbing was mediated by materialism, supporting H9 (indirect-only/full mediation). This finding provides a theoretically meaningful explanation for why attachment avoidance did not show a clear direct association with enacted phubbing (H3) in the correlational analysis, yet still contributed to enacted phubbing through a motivational mechanism. Specifically, avoidant attachment may not directly elicit enacted phubbing as a distancing strategy, but may increase materialistic striving, which then promotes smartphone engagement in interpersonal contexts.

With respect to perceived phubbing, the relationship between attachment anxiety and perceived phubbing was significantly mediated by materialism, confirming H11 (partial mediation). This result suggests that attachment anxiety increases perceived partner phubbing both through direct threat sensitivity (H1) and through materialistic value orientation. One interpretation is that materialism may heighten sensitivity to social comparison cues and external evaluation, thereby amplifying perceived relational threats when a partner attends to a smartphone.

Finally, the relationship between attachment avoidance and perceived phubbing was mediated by materialism, supporting H12 (indirect-only/full mediation). Together, these mediation findings support the proposition that attachment insecurity influences phubbing both directly and indirectly, and that materialism constitutes a central connecting mechanism linking relational insecurity with both behavioral and perceptual forms of phubbing.

The findings further contribute to theory by illustrating that attachment anxiety and attachment avoidance are linked to phubbing through partially distinct psychological pathways. Attachment anxiety was consistently associated with higher levels of enacted and perceived phubbing, both directly and indirectly via materialism. This pattern aligns with the hyperactivating strategies characteristic of anxious attachment, whereby individuals remain vigilant to alternative sources of reassurance and validation. In digital contexts, this heightened sensitivity may foster increased engagement with smartphones and a stronger tendency toward enacted phubbing as a means of managing relational uncertainty.

Attachment avoidance, i contrast, showed weaker direct associations but meaningful indirect effects through materialism (cf., H9 and H12). This suggests that avoidantly attached individuals may not engage in phubbing primarily due to interpersonal dependency concerns, but rather because materialistic orientations support distancing strategies and emotional self-sufficiency. Phubbing may thus function as a socially acceptable form of disengagement that aligns with deactivating attachment strategies, allowing avoidant individuals to regulate closeness without overt interpersonal conflict.

Together, these differentiated pathways emphasize that phubbing is not a uniform behavior driven by a single motivational process, but rather reflects distinct attachment-related regulation strategies that converge on similar observable outcomes.

### 4.5. Integration with Existing Models of Phubbing and Social Media Use

The present findings also contribute to existing theoretical perspectives on smartphone-related interpersonal behavior. Phubbing has been linked to reciprocity and social norms, as well as to fear of missing out, low self-control, and problematic smartphone use (Chotpitayasunondh & Douglas, 2016; Schneider & Hitzfeld, 2021). Our results complement these accounts by highlighting a more distal personality-based foundation for phubbing, rooted in attachment-related insecurity and value-based motivation.

Specifically, materialism may represent a motivational bridge between attachment insecurity and more proximal mechanisms commonly discussed in phubbing research, such as FoMO. It is plausible that materialism enhances FoMO because materialistic individuals may be especially oriented toward what others possess, experience, or display in the digital environment. In turn, FoMO could instigate enacted phubbing by increasing the perceived urgency of checking updates, monitoring social information, or maintaining digital connectedness. Empirical evidence supports that materialism and FoMO are positively correlated (Long et al., 2021). Thus, while FoMO was not directly assessed here, future research may profit from extending the present model by incorporating FoMO as an additional mediator or proximal mechanism.

Overall, the present study advances the theoretical framework of phubbing by integrating attachment processes (social bonding and threat regulation) with materialism (identity and status-oriented goal pursuit) and by demonstrating their combined relevance for enacted and perceived phubbing.

Future research should further examine boundary conditions and situational moderators that determine when attachment insecurity translates into enacted phubbing. In particular, dyadic designs and daily diary studies could clarify whether enacted phubbing reflects deliberate disengagement, reassurance seeking, or habit-based smartphone checking depending on interaction context. Moreover, extending the present model by including problematic smartphone use, FoMO, and relationship satisfaction may help to specify whether attachment insecurity and materialism influence phubbing primarily through motivational processes, self-regulatory deficits, or relational meaning-making.

Beyond their theoretical relevance, the present findings also have important practical implications for relationship counseling, digital well-being interventions, and prevention efforts. First, the results suggest that phubbing should not be addressed solely as a problematic habit of smartphone overuse, but as a behavior that is meaningfully embedded in relational dynamics and value orientations. Interventions that focus exclusively on reducing screen time may therefore overlook important psychological drivers.

Second, the differentiated roles of attachment anxiety and avoidance imply that intervention strategies should be tailored to underlying attachment-related needs. For individuals high in attachment anxiety, interventions may benefit from strengthening perceived relational security, fostering emotion regulation skills, and reducing reliance on external validation through materialistic or digital sources. For avoidantly attached individuals, therapeutic approaches might focus on increasing awareness of distancing strategies, promoting engagement in face-to-face interactions, and addressing materialistic beliefs that reinforce emotional disengagement.

Finally, the central mediating role of materialism suggests that value-oriented interventions may be particularly effective. Addressing materialistic value orientations—by fostering intrinsic goals, relational mindfulness, and awareness of how digital devices are used to compensate for insecurity—may indirectly reduce both enacted and perceived phubbing. From this perspective, reducing phubbing is not only a matter of changing behavior, but also of reshaping the underlying meanings attributed to digital objects in social relationships.

## 5. Limitations

Several limitations should be noted. First, women (72.3%) and students (73.2%) were overrepresented in the sample, which restricts the generalizability of the findings; future studies should therefore recruit more male and non-student participants. Second, the study relied primarily on self-report measures, which may be influenced by response styles such as acquiescence and socially desirable responding, potentially leading participants to underreport materialism as well as attachment anxiety and avoidance; future research should therefore incorporate additional behavioral or observational indicators of phubbing. Third, although the G-MVS has demonstrated convergent and discriminant validity (e.g., positive associations with compulsive buying and no association with depression), the present study design remains correlational and cross-sectional, which precludes conclusions about temporal precedence and causal pathways. Accordingly, the proposed mediation processes should be examined using longitudinal, dyadic, or experimental designs to clarify directionality and establish causal inferences.

## 6. Conclusions

This study advances phubbing research by integrating attachment theory with a materialism-based perspective. The findings indicate that insecure attachment is linked to both enacted and perceived phubbing and, crucially, that materialism serves as a key mechanism through which attachment-related insecurities translate into smartphone-related relational disruption. In this sense, phubbing appears not only as a behavioral byproduct of mobile technology use, but also as a psychologically meaningful expression of how individuals regulate insecurity and pursue external validation in close relationships. Targeting materialistic value orientations and attachment-related vulnerabilities may therefore represent promising avenues for reducing phubbing and strengthening relationship functioning.

## Figures and Tables

**Table 1:** Intercorrelations of the variables.

	1	2	3	4	5
1 Attachment anxiety	-	−0.16 *	0.37 ***	0.36 ***	0.26 ***
2 Attachment avoidance		-	0.18 **	0.09	0.14 *
3 Materialism			-	0.42 ***	0.24 ***
4 Enacted phubbing				-	0.22 ***
5 Perceived phubbing					-

Note. df = 213; * *p* < 0.05; ** *p* < 0.01; *** *p* < 0.001.

## Data Availability

The dataset generated and analyzed during the current study will be made available in the OSF repository. The link will be shared—because of the double-blind peer review process—after acceptance of the paper.

## Appendix A. Translation of the Phubbing Scale

**Table A1:** The German Version of the Phubbing Scale.

	Original Items	German Version
1.	My eyes start wandering on my phone when I’m together with others.	*Wenn ich mit anderen zusammen bin, beginnt mein Blick zu meinem Handy zu wandern.*
2.	I am always busy with my mobile phone when I’m with my friends.	*Ich bin immer mit meinem Handy beschäftigt, wenn ich mit meinen Freund/innen zusammen bin.*
3.	People complain about me dealing with my mobile phone.	*Andere beschweren sich darüber, dass ich mich mit meinem Handy befasse.*
4.	I’m busy with my mobile phone when I’m with friends.	*Ich bin mit meinem Handy beschäftigt, wenn ich mit Freund/innen zusammen bin.*
5.	I don’t think that I annoy my partner when I’m busy with my mobile phone.	*Ich denke nicht, dass ich meine/n Partner/in damit nerve, wenn ich mit meinem Handy beschäftigt bin.*
6.	My phone is always within my reach.	*Mein Handy ist immer in Reichweite.*
7.	When I wake up in the morning, I first check the messages on my phone.	*Wenn ich morgens aufwache, checke ich zuerst die Nachrichten auf meinem Handy.*
8.	I feel incomplete without my mobile phone.	*Ohne mein Handy fühle ich mich unvollständig.*
9.	My mobile phone use increases day by day.	*Meine Handy-Nutzung wird von Tag zu Tag mehr.*
10.	The time allocated to social, personal or professional activities decreases because of my mobile phone.	*Die Zeit, die ich sozialen, persönlichen oder beruflichen Aktivitäten widme, nimmt aufgrund meiner Handy-Nutzung ab.*

Note. Items are answered on a Likert scale from 1 (never) to 5 (always).

**Table A2:** The German Version of the Partner Phubbing Scale.

	Original Items	German Version
1.	During a typical mealtime that my partner and I spend together, my partner pulls out and checks his/her cell phone (slight modification).	*Während einer typischen Mahlzeit, die mein/e Partner/in und ich gemeinsam verbringen, holt mein/e Partner/in sein/ihr Handy raus und überprüft es.*
2.	My partner places his or her cell phone where they can see it when we are together.	*Wenn wir zusammen sind, platziert mein/e Partner/in sein/ihr Handy so, dass er/sie es sehen kann.*
3.	My partner keeps his or her cell phone in their hand when he or she is with me.	*Mein/e Partner/in behält sein/ihr Handy in der Hand, wenn er/sie mit mir zusammen ist.*
4.	When my partner’s cell phone rings or beeps, he/she pulls it out even if we are in the middle of a conversation (slight modification).	*Wenn das Handy meines Partners/meiner Partnerin klingelt oder piept, holt er/sie es heraus, auch wenn wir mitten im Gespräch sind.*
5.	My partner glances at his/her cell phone when talking to me.	*Mein/e Partner/in schaut auf sein/ihr Handy, während er/sie mit mir spricht.*
6.	During leisure time that my partner and I are able to spend together, my partner uses his/her cell phone (slight modification).	*In der Freizeit, die mein/e Partner/in und ich zusammen verbringen können, nutzt mein/e Partner/in sein/ihr Handy.*
7.	My partner does not use his or her phone when we are talking.	*Mein/e Partner/in nutzt sein/ihr Handy nicht, wenn wir uns unterhalten.*
8.	My partner uses his or her cell phone when we are out together.	*Mein/e Partner/in nutzt sein/ihr Handy, wenn wir gemeinsam unterwegs sind.*
9.	If there is a lull in our conversation, my partner will check his or her cell phone.	*Gibt es eine Pause in unserem Gespräch, checkt mein/e Partner/in sein/ihr Handy.*

Note. Items are answered on a Likert scale from 1 (“Never”) to 5 (“All the time”).
